# Opioid agonist treatment (OAT) experiences and release plans among federally incarcerated individuals with opioid use disorder (OUD) in Ontario, Canada: a mixed-methods study

**DOI:** 10.1186/s12889-022-12685-0

**Published:** 2022-03-04

**Authors:** Cayley Russell, Frishta Nafeh, Michelle Pang, Shanna Farrell MacDonald, Dena Derkzen, Jürgen Rehm, Benedikt Fischer

**Affiliations:** 1 Institute for Mental Health Policy Research (IMHPR), Centre for Addiction and Mental Health (CAMH) & Canadian Research Initiative in Substance Misuse (CRISM), 33 Ursula Franklin St, Toronto, ON M5S 2S1 Canada; 2 Research Branch, Correctional Service Canada, 340 Laurier Avenue West, Ottawa, ON, K1P 0P9 Canada; 3 Department of Psychiatry, University of Toronto, 1 King’s College Circle, Toronto, ON M5S 1A8 Canada; 4 Dalla Lana School of Public Health, University of Toronto, 155 College St, Toronto, ON M5T 3M7 Canada; 5 Campbell Family Mental Health Research InstituteCentre for Addiction and Mental Health, Toronto, ON, M5S 2S1 Canada; 6 Institute of Medical Science (IMS), University of Toronto, 1 King’s College Circle, Toronto, ON M5S 1A8 Canada; 7 Institut für Klinische Psychologie und Psychotherapie, Technische Universität Dresden, Chemnitzer Str. 46, 01187 Dresden, Germany; 8 Department of International Health Projects, Institute for Leadership and Health Management, I.M. Sechenov First Moscow State Medical University, Bol’shaya Pirogovskaya Ulitsa, 19с1, Moscow, Russia 119146; 9 Centre for Applied Research in Mental Health and Addiction (CARMHA), Faculty of Health Sciences, Simon Fraser University, Vancouver, BC, V6B 5K3 Canada; 10 School of Population Health and Pharmacy, Faculty of Medical and Health Sciences, University of Auckland, Grafton, Auckland, 1023 New Zealand; 11 Department of Psychiatry, Federal University of São Paulo (UNIFESP), São Paulo, Brazil

**Keywords:** Canada, Community-release, Federal Correctional Institutions, Health services, Opioids, Opioid agonist treatment, Opioid use disorder, Prison, Methadone, Buprenorphine-naloxone (Suboxone)

## Abstract

**Background:**

Incarcerated populations experience an elevated prevalence of opioid use disorder (OUD). Federal correctional institutions in Canada have increasingly treated OUD among correctional populations via opioid agonist treatment (OAT) – an evidence based pharmacotherapy that works to reduce drug use and related health harms. However, there is limited evidence regarding incarcerated individuals’ experiences with institutional-based OAT, as well potential OAT-related community release prospects. This information is important for optimal treatment retention and improved health. To address this knowledge gap, we conducted a longitudinal follow-up study examining OAT-related experiences among federally incarcerated individuals before and after community release. This article focuses on the baseline (pre-release) data.

**Methods:**

This mixed-methods study examined OAT-related experiences and release prospects among *n* = 46 individuals scheduled for community release, recruited from seven federal prisons located in Ontario, Canada. Participants underwent a comprehensive interviewer-administered on-site assessment, including quantitative and qualitative items. Assessment data was furthermore linked to administrative correctional data. Data were analyzed using thematic qualitative and descriptive quantitative approaches.

**Results:**

Participants had complex histories with opioid use including related negative health outcomes. Experiences with institutional OAT were divergent and provision was not standardized; those with OAT engagement pre-admission did not experience many challenges, whereas those initiating OAT during incarceration experienced barriers such as treatment waitlists and adverse process experiences. Most participants expressed a preference for buprenorphine-naloxone over methadone, but described difficulties accessing it. Participants were keen to transition into community-based treatment, yet envisaged prospective barriers and facilitators concerning successful reintegration and treatment continuity.

**Conclusions:**

Major barriers towards the current administration of OAT in federal correctional systems in Canada exist, including extensive waitlists, non-standardized practices, and challenges accessing preferred OAT formulations; this contributes to sub-optimal treatment. Eliminating waitlists, standardizing OAT provision, providing additional OAT options, and more comprehensive release planning may be essential for treatment retention and positive outcomes.

## Background

Correctional populations experience an elevated prevalence of substance use-related issues, including problematic opioid use and opioid use disorder (OUD) [1–4]. Globally, substance use disorders among correctional populations have been estimated at 30 and 51% among males and females, respectively [1]. Despite a zero-tolerance policy for substance use in correctional institutions, substance use remains prevalent during incarceration periods, with global rates estimated around 20–40% [5–7]. Specifically, in Canada, over 70% of a sample of federally incarcerated men reported having a substance use problem and 16% reported being under the influence of opioids on the day of their offence between 2006/07–2008/09 [8–10]. Similarly, four-fifths of federally incarcerated women reported a substance use problem at the time of admission, and these rates were even higher among Indigenous women, likely due in part to the longstanding negative impacts of colonialism [11–14]. Other Canadian data confirm high rates of opioid use prior to and during incarceration, as well as a high prevalence of OUD diagnoses among correctional populations [11, 15–18]. Further, OUD within Canadian correctional facilities has been increasingly linked with adverse outcomes including overdose incidents [19]. For instance, a total of 330 overdoses occurred within federal correctional institutions between 2012/2013 and 2016/2017; of the 7% of overdoses which were fatal, over 90% were due to opioids, with 36% involving fentanyl [20].

Within the community and correctional settings, the primary treatment for OUD in Canada is opioid agonist treatment (OAT), including methadone and/or buprenorphine-naloxone (Suboxone) formulations. OAT is an evidence-based, safe, and effective treatment for OUD which reduces withdrawal symptoms and opioid use and related risks [21–25]. Canadian clinical guidelines now recommend buprenorphine-naloxone-based OAT as the first-line treatment for OUD, while federal correctional institution OAT policies refer to these guidelines as a standard [20, 21], and international human rights guidelines emphasize equal rights for individuals to be provided with the same healthcare (including OAT) as received in the community [26–30]. However, historically, the implementation and uptake of OAT programs in correctional settings has been restricted, and a variety of structural, cultural, and organizational barriers have rendered them far limited and inferior in quality compared to community-based OAT [29, 31, 32]. For example, correctional institutions often face qualified healthcare staff shortages, and institutional policies typically focus on security concerns and abstinence-based treatment approaches based on traditional tenets of punishment and risk-reduction over health interventions, including penalization of individuals for drug use during incarceration [31, 33].

Thus, the provision of OAT in correctional settings remains limited. In the United States, only 40 out of over 5000 local/county jails or state/federal prisons offered OAT as of 2016, rendering the vast majority of incarcerated individuals with OUD unable to access a vital evidence-based treatment and leaving them vulnerable to withdrawal and other adverse events [34–38]. In Canada, correctional OAT demand and coverage has substantially increased over recent years; OAT is now available in all federal prisons and most provincial/territorial correctional settings [29, 39]. For instance, Correctional Service Canada (CSC) (the Canadian federal government agency responsible for administering criminal sentences of 2 years or more to incarcerated adults), has provided methadone to federally incarcerated individuals with OUD for over 20 years, further expanding the program to offer buprenorphine-naloxone in 2008 [40–44]. The total number of OAT patients across federal correctional institutions has more than doubled from 920 in 2016, to 2481 in January 2021, and approximately 15% of all federally incarcerated individuals now receive OAT [45, 46]. OAT formulation options have recently expanded to include different buprenorphine-naloxone preparations (e.g., sublingual tablet and/or sublingual/buccal film) as well as extended-release injectable buprenorphine [Sublocade] [20, 46].

OAT is associated with a number of positive outcomes among correctional populations with OUD, such as increases in drug treatment entry and retention, reductions in drug – and opioid – use, as well as reduced recidivism, overdose, and mortality [35, 47–50]. Specifically, a recent systematic review found that methadone treatment initiated during incarceration is associated with significantly higher post-release community-based OAT engagement, and reductions in illicit opioid use and injection drug use [49]. In Canada, correctional OAT involvement has been associated with a decreased likelihood of overdose and non-medical prescription opioid use [51], as well as a lower risk of returning-to-custody [11], and lower rates of violent and non-violent offences [52]. Moreover, a correctional health care service evaluation (2017) found that individuals participating in CSC’s OAT program reported a lower prevalence of injection drug use, needle-sharing, positive urinalysis tests, serious disciplinary offences, and an increase in educational program participation during incarceration [53].

These statistics underscore both the importance of providing OAT to correctional individuals with OUD, and the need for effective transition of inter-institutional (e.g., correctional transfers) and community-based OAT care after release; the latter has been identified as a crucial point where OAT care (e.g., retention) is commonly compromised or interrupted [54–59]. Furthermore, correctional populations face high odds of substance use-related relapse and overdose upon release, which can be mediated by OAT provision [48, 60]. Given the high rates of OUD among correctional populations, and high proportion of individuals receiving OAT in CSC, OAT is recognized as an essential health care intervention, yet with considerable risk for continued substance use, relapse, and overdose risk both during and post-incarceration if not provided effectively and/or continuously [51]. The continuous provision of OAT to incarcerated individuals with OUD is therefore especially pertinent, given these adverse health risks as a possible outcome of ineffective OAT care. However, there is a lack of information on individuals' experiences with OAT care during incarceration and community release prospects and related factors which may influence outcomes, especially in the Canadian context. In order to address this knowledge gap, we conducted the present study with a cohort of federally incarcerated individuals receiving OAT care and scheduled for community release across institutions in Ontario, Canada.

## Methods

### Study design

This paper focuses on the *baseline* results of a longitudinal, mixed-methods observational study examining OAT-related experiences among a multi-institutional sample of federally incarcerated individuals. Two separate assessments were conducted, occurring at two separate time points: during the sample’s 1) pre-release incarceration period (baseline); and 2) community transition period following-release (follow-up). The study was conducted independently by an external academic team and field researchers, with logistical support from CSC.

### Eligibility

Study eligibility criteria included individuals who were: 1) currently incarcerated at one of the seven Ontario-based CSC institutions; 2) diagnosed with OUD as per clinical requirement to receive institutional OAT care; 3) involved in CSC’s OAT program for at least 3 months; 4) given a statutory release or parole eligibility date scheduled within 6 months of the baseline (pre-release) interview; 5) with an expected release location within Ontario; and 6) willing and consenting to participate in both the study’s baseline (pre-release) and follow-up (post-release) assessments.

### Recruitment and consent

Participant recruitment and institutional access/assessment space was facilitated with the assistance of institutional site contacts at each CSC institution. An up-to-date list of individuals on OAT with release dates scheduled within the next 6 months was provided by CSC to institutional site contacts (e.g., healthcare administrators) at each institution. These site contacts then approached the individuals listed and provided them with a study flyer that included a toll-free study line allowing individuals to anonymously contact the research team if they were interested in participating. The study flyer was also openly posted in the healthcare unit for anyone to see and follow-up with the research team if interested. Further, individuals also had the option of expressing interest to the study staff directly for study participation screening procedures.

The research team (two field researchers) arranged approximately week-long field visits at each CSC institution for study participant recruitment and assessments. Upon arrival, individuals who had expressed interest in participating met with the researchers to be screened for eligibility where they provided their federal fingerprint serial (FPS) number, as well as a pseudonym, and were given a study ID number to identify them on all study documents. The eligibility screening consisted of a brief 8-question protocol. Upon determination of an individual’s eligibility, the research team then scheduled participants to complete the main assessment at a pre-determined time (aligned with institutional schedules) during the same institutional research visit.

Participants were informed that the study was being confidentially conducted by an external research group (i.e., not affiliated with CSC) without individual study information being shared with anyone outside the research team, including any CSC personnel. No personal identifying information was collected; participants’ FPS numbers were the only identifiers used to link administrative data from CSC databases, and the pseudonym participants provided (and corresponding study ID given) was used on all subsequent study documents and analysis. Informed written consent was obtained from all participants, for both the baseline and follow-up assessments, for explicit linkage of individual administrative data from CSC databases, as well as for future contact with the participant and their community parole officer to facilitate the follow-up assessment. Study participants were offered a $50 honoraria upon completion of the follow-up assessment (as honoraria could not be provided during incarceration as per CSC guidelines) [61].

### Data collection and assessment tools

Between January 15th and March 31st, 2019, the research team visited each of the participating seven (6 men, 1 women) federal CSC institutions located across Ontario (see Appendix 1, Fig. 2 for a map and institutional security levels) to conduct the baseline (pre-release) assessments with study participants. Data collection was completed over the course of approximately 1 week per institution, and each assessment was completed in a single session (approximately 45–90 min in time). All assessments were conducted in a private interview room with security arrangements; study participants were provided with a ‘pass’ which allowed them to attend the assessment at the time scheduled.

The baseline assessment consisted of a quantitative (pen-and-paper), interviewer-administered survey, followed by a qualitative (semi-structured, audio-recorded, one-on-one interview-based) component. The survey took approximately 15–30 min to complete. The data timeframe included a primary focus on two temporal snapshots: 1) ‘30 days prior to incarceration’ and 2) ‘past-30 days’ (during incarceration). The qualitative interview took approximately 30–60 min and consisted of 9 open-ended principal questions (see Appendix 2 for interview guide).

Additionally, using participants’ FPS numbers, we linked select complementary aggregate administrative data, mainly comprising basic socio-demographic, criminogenic and institutional-behavioral data for each participant, extracted and provided from two primary CSC-based databases: the Offender Management System (OMS) which maintains all individual records and data (e.g., socio-demographics) [62]; and the Computerized Assessment of Substance Abuse (CASA) database which documents substance use among individuals admitted to federal custody [63–65]. The CASA assessment is based on ratings obtained via three standardized tests, and is a standardized, computer-assisted, self-administered assessment completed by all individuals admitted to federal custody during the intake process. These data variables were stripped of any identifying information and shared via an encrypted file transfer using participants’ FPS numbers.

### Data processing and analysis

Hardcopy survey documents were stored in a locked filing cabinet, and all survey data were entered into an encrypted excel database for data processing and analysis. Basic descriptive statistics (e.g., mean and frequency values) were performed on participant characteristics and behavioral data, using a combination of participant survey and select complementary administrative CSC data.

All audio-recorded interviews were transcribed verbatim and imported into qualitative data management software (NVivo 12). Audio recordings were deleted subsequent to transcription. Any potentially identifying information that may have been revealed during the interview process (e.g., references to city or service names, etc.) were removed from the data. Qualitative interview data underwent an inductive thematic analysis process, whereby key initial themes were identified, and a preliminary codebook was developed in Excel [66]. Specifically, one member of research team (CR) open-coded the transcripts based on the initial codebook to identify common responses to the study’s research questions. Following extensive discussion among the research team, the initial codes were then refined and applied to the data. Additional codes emerging from the data were subsequently added to the codebook as part of the iterative coding process [67]. In order to ensure transparency and consistency in data analysis, the research team utilized inter-coder reliability whereby an independent coder (FN) coded a randomly selected sub-sample (20%) of the transcripts [68], and any codebook revisions and coding queries were resolved with the team based on ongoing discussion. The final qualitative themes and sub-themes presented were informed by multiple participants conveying similar sentiments and statements until data saturation was met [69, 70]. All themes were narratively summarized for results and are further illustrated and substantiated by select participant quotes.

### Ethics

Study procedures were approved by the Centre for Addiction and Mental Health (CAMH) Research Ethics Board (REB: #013–2018).

## Results

### Sample

A total of *n* = 68 potentially eligible individuals were screened for participation across the seven CSC institutions; *n* = 22 participants were not included as they either were ineligible, not interested, or could not attend their scheduled assessment. The final study sample included *n* = 46 (40 men and 6 women) participants. The following briefly summarizes variables from the baseline survey data in conjunction with administrative CSC data.

### Quantitative results

#### Socio-demographics (see Table 1)

The sample’s mean age was 36.4 years; the majority (87%) were men who reported their ethno-cultural/racial background as white (65%); 33% reported identifying as Indigenous. Most (65%) participants had less than high school education. Prior to incarceration, 54% reported having unstable accommodation, and the majority (83%) participated in illegal activities (e.g., selling drugs, robberies, debt collecting, other income generation crimes, etc.) as their main source of income.

**Table 1:** Sociodemographic characteristics of study sample (*N* = 46)

Characteristic	% (***n***)
**Gender**	
Men	87% (40)
Women	13% (6)
**Age (**Mean ± SD)	36.4 ± 7.7
**Race**^b^	
White/other	67% (31)
Indigenous	33% (15)
**Less than high school diploma**^b^	65% (30)
**Unstable accommodation prior to incarceration**^b^	54% (25)
**Main source of income (30 days prior to incarceration)**^a^	
Illegal activities	83% (38)
Social assistance	61% (28)
Family/friends	33% (15)
Legal employment	26% (12)
Illegal work	26% (12)
Personal savings	17% (8)
Other	9% (4)

^a^Responses not mutually exclusive

^b^Data acquired from CSC. CSC collects data on distinctions between First Nations, Métis, and Inuit, but due to the small sample size we have kept these categorized as Indigenous/non-Indigenous

#### Criminogenic and institutional-behavioral characteristics (see Table 2)

Participants’ mean sentence length was 3.1 years, and 39% were serving a second or subsequent sentence. The most common offence type for which participants were serving time was ‘violent’ (35%), followed by ‘drug-related’ (22%). Nearly half (44%) had a history of institutional charges and 22% had an institutional incident record related to drug contraband. Nearly one in five (17%) had a positive illicit substance urinalysis test result at some point during their incarceration period, with 8% positive for opioids.

**Table 2:** Criminogenic and institution-specific characteristics of study sample (*N* = 46)

Characteristic	% (***n***)
**Security level at interview**	
Medium/maximum^b^	78% (36)
Minimum	22% (10)
**Offence type**^a^	
Violent	35% (16)
Drug-related	22% (10)
Robbery	22% (10)
Property-related	15% (7)
Non-violent	7% (3)
**Sentence length (years)**^a^	
*Mean ± SD*	3.1 ± 1.3
**Number of sentences**^a^	
1	61% (28)
2+	39% (18)
**Institutional activities**^a c^	
Correctional Programming	83% (38)
Education	39% (18)
Employment	20% (9)
**History of institutional charges**^a c^	44% (20)
**Institutional incidents**^a c^	46% (21)
Drug contraband	22% (10)
**Institutional urinalysis**^ac^	78% (36)
Positive for illicit drug use	17% (6)
Positive for illicit opioid use	8% (3)

^a^Data acquired from CSC; Violent offences include homicide, sex-related, assault, and other

^b^Only one participant had a maximum security classification

^c^Timeframe was between initial admission to federal custody and interview date. Correctional programming entails multi-target skill-based learning programs that address multiple risk factors linked to individuals’ criminal behaviour, offered at moderate and high-risk intensity levels

#### Substance use and treatment (see Table 3 and Fig. 1)

Most (84%) participants had a ‘substantial’ or ‘severe’ substance use problem assessed at admission. Half (50%) reported both a lifetime, and recent pre-incarceration history of injection drug use. About half (44%) were engaged in OAT pre-incarceration with almost all receiving methadone-based OAT. Methadone was also the most commonly used institutional OAT formulation (67%). Over half (57%) had a history of polysubstance use; stimulants (78%), prescription opioids (78%), and heroin/other illegal opioids (67%), were the most commonly used drugs pre-incarceration, while cannabis use (28%) was the most common during incarceration.

**Table 3:** Substance use and treatment characteristics of study sample (*N* = 46)

Characteristic	% (***n***)
**Severity of substance use at admission**^b^	
Moderate/Severe^d^	84% (36 of 43)
**History of polysubstance use**^b^	57% (26)
**History of injection drug use**	50% (23)
Lifetime^b^	50% (23)
30 days prior to incarceration	37% (17)
**Non-OAT substance use services used (30 days prior to incarceration)**^a^	
Harm reduction services	46% (21)
Outpatient treatment	17% (8)
Support groups	15% (7)
Inpatient treatment	9% (4)
**Engagement in OAT (30 days prior to incarceration)**	44% (20)
**Type(s) of OAT (30 days prior to incarceration)**^ac^	
Methadone	95% (19 of 20)
Buprenorphine-naloxone	5% (1 of 20)
Both Methadone and buprenorphine-naloxone	10% (2 of 20)
Hydromorphone	5% (1 of 20)
**Entered CSC on OAT**	39% (18)
**Type(s) of OAT during CSC incarceration**^b^	
Methadone	67% (31)
Buprenorphine-naloxone	33% (15)

^a^Responses not mutually exclusive

^b^Data acquired from CSC

^c^Responses out of *n* = 20 participants who indicated they had been engaged in OAT 30 days prior

^d^Responses out of *n* = 43 participants who had this data; Severity of substance use is determined using CASA. Harm reduction category includes use of either needle exchange, safer use kits, naloxone kits, etc.; Outpatient treatment category includes either group therapy, one-on-one therapy/counseling, relapse prevention; Inpatient treatment category includes residential treatment, rehabilitation, detoxification/withdrawal management; Support groups include self-help or mutual aid groups such as alcoholics anonymous, narcotics anonymous, etc.

**Fig. 1:**
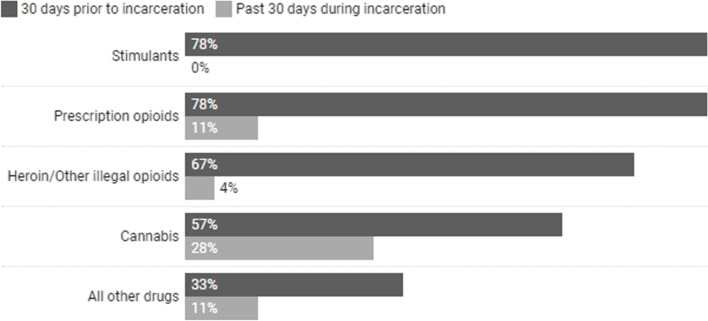
Substance use among study participants (*N* = 46). Note: Substance use is not mutually exclusive. Stimulants include use of either methamphetamines/amphetamines, cocaine, or crack-cocaine. Prescription opioids include use of either codeine, hydrocodone, tramadol, morphine, hydromorphone, meperidine, oxycodone, fentanyl, or methadone/buprenorphine. Prescription opioids were not necessarily used as prescribed, and use could have been non-medical. Other illegal opioids include synthetic or adulterated prescription opioids. All other drugs include use of either hallucinogens, benzodiazepines, or other psychotropic drugs (e.g., antidepressants, antipsychotics)

### Qualitative results

Qualitative data results are presented under the following overarching themes: opioid use initiation and trajectories; opioid-related health effects; benefits of OAT; OAT formulation preferences; diverse experiences accessing institutional OAT; institutional and physician discrepancies; potential barriers to community reintegration; and potential facilitators to community reintegration.

#### Opioid use initiation and trajectories

All participants reported experiences with opioid use prior to incarceration, and many indicated that they commonly engaged in polysubstance use. While the majority of participants described experimenting with drugs (including opioids) during adolescence or early adulthood, just under half reported being introduced to opioids through legitimate prescriptions, commonly for pain management and/or other medical reasons. Most of these participants suggested that they had been unaware of the addictive potential of opioids, and that they had been prescribed pharmaceutical opioids for a period of time before their use patterns changed. The trajectory of opioid use for many accelerated after tolerance increased and/or their provider stopped prescribing them. As such, many reported increasing their use and/or switching to illicitly-sourced opioids for access, affordability, or the need to utilize more potent opioids:

*“I threw my back out and it started with doctors giving me [Percocet], and it just progressed from there … I got cut off from the Percs from my doctor, so I turned to the streets for them … I started taking Oxy 10’s, 20’s, 40’s, and just 80’s.” (Participant 35)*

Similarly, progressive changes in route of administration were reported by most respondents. The majority had started with oral use and over time graduated to inhalation, and in some instances, injection use of opioids in order to achieve desired effects.

#### Opioid-related health effects

Most respondents indicated that their opioid use caused various physical harms, such as track (injection) marks, abscesses, dental, and weight-related problems, as well as drowsiness, tolerance, dependence, and withdrawal. In some cases, participants contracted infectious diseases such as Hepatitis C from sharing drug paraphernalia:

*“I did obtain Hep C. However, I took the treatment when I was here, on my first federal bit. I got out and I did inject again, because I relapsed … but if you were to take a blood sample you would see that I have the antibodies, but I’m not contagious.” (Participant 28)*

While many participants described witnessing friends, peers, or family members experience overdoses, a minority indicated they had personally experienced opioid overdoses (within the community and/or during incarceration).

In many cases, participants suggested that opioids had also impaired their mental well-being, citing symptoms of memory loss, hallucinations, depression, anxiety, and/or suicidal thoughts. Several respondents also reported having co-morbid mental health symptoms/diagnoses, some of which may have been exacerbated by their opioid use:

*“I attempted suicide in the past year four times. I’m not saying that just to get sympathy, but yeah, I think it has [impacted my mental health] to a certain degree. I’ve only recently developed bipolar disorder … maybe that’s the impact that the drugs have, I don’t know. I have PTSD.” (Participant 42)*

#### Benefits of OAT

The majority of participants had a history of pre-incarceration engagement in OAT. While just under half were OAT-involved immediately prior to incarceration, others had gone on-and-off OAT for several years, both within the community and during different incarceration periods. Regardless of their OAT history, most respondents suggested that overall, OAT had been beneficial and helped them avoid withdrawal symptoms, temper substance use cravings and triggers, as well as reduce their opioid use:

*“Some of my urges, like cravings, are just gone, and when I do some of the things that triggered me before, they don't trigger me anymore. When I do get triggered, I have more control over the cravings.” (Participant 36)*

Specific benefits included the structure that OAT provided in participants’ lives by giving them a daily purpose and routine. Many indicated that having to consistently provide negative urinalysis tests gave them incentive to refrain or reduce their substance use:

*“Yeah, like I said, it puts structure in my life. The weekly urinalysis and that, like, really, is what mostly did it for me. ‘Cause like, I got embarrassed about giving dirty urines. You know, going in there asking for help and then just constantly giving dirty urines. So that’s something that’s really put me on the straight and narrow.” (Participant 04)*

#### OAT formulation preferences

Preferences for buprenorphine-naloxone versus methadone-based OAT varied among participants. The majority reported that while methadone was better known and more utilized since it had been available for longer, they preferred buprenorphine-naloxone. These participants explained that they not only experienced fewer long-term side effects from buprenorphine-naloxone, but that they were also under the impression that it safeguarded against using other opioids due to its abuse-deterrent formulation (i.e., the naloxone component). Conversely, a few participants explained that since once can use other opioids while on methadone, it was used by some to reduce opioid use and consequent spending, and to avoid related withdrawal symptoms:

*“While I was on methadone, I was still using heroin. I was, like, literally still using it. So it kind of was helping, but at the same time, it wasn’t. Methadone really wasn’t working for me while I was in the community. I kind of gave up on it and just said, forget it, and I continued to do drugs. The fact that you can drink methadone and still do drugs, it’s not helping. Whereas, like, with the Suboxone, I was told that if you use Suboxone and do drugs that you go into immediate withdrawal.” (Participant 12)*

Additionally, most participants described methadone as being associated with more stigma and having worse health effects such as dental decay, weight gain, low testosterone/decreases in libido, lethargy, organ (e.g., liver and kidney) problems, and headaches. In addition, many perceived methadone to cause more severe withdrawal and dependence symptoms, describing it as ‘liquid handcuffs’ or stating that they could ‘feel it in their bones’. It was suggested that ceasing or weaning off of it was extremely difficult due to the physical effects:

*“Methadone was, I think, harder to come off with than any other drug. Harder to come off with than Suboxone. I’ve done Suboxone while I was in the provincial system. I was doing it every day for about thirty days, I was buying it off another inmate. But even coming off of that was nothing compared to coming off of Methadone. Coming off of Methadone was worse than coming off heroin, oxycontins, percs, fentanyl. Yeah, it was bad.” (Participant 12)*

#### Diverse experiences accessing institutional OAT

In terms of receiving institutional OAT, two overarching and divergent experiences were expressed. One the one hand, participants who were engaged in OAT immediately pre-incarceration commonly described an easy and relatively seamless transition from community-based into institutional-based OAT. Participants only had to prove when they had taken their last dose (e.g., present paperwork or confirmation with their community doctors), which typically took anywhere from 1 day up to 2 weeks. This was the case for about half of the participants with prior OAT engagement, during which time they experienced treatment interruptions and related adverse effects, including withdrawal:

*“It was pretty horrible actually. When I had gotten arrested I had just gotten my carries [take-home OAT] and they were all at my house. And so they wouldn't give me any methadone for, like, I think it was almost two weeks before I got my methadone again. It was horrible.” (Participant 21)*

Conversely, those who were not on community-based OAT immediately prior to their current incarceration (i.e., just over half of participants), or who had experienced medication disruptions at the provincial correction point-of-entry to the criminal justice system, experienced significant challenges in accessing CSC’s OAT program. These participants reported extensive wait times (in most instances amounting to multiple months) before they were able to enroll in institutional OAT. This was for several reasons, including participants being housed at assessment institutions where they were not allowed to initiate OAT until transferred to their main institution. Other reasons included institutional policies which stipulated maximum numbers of OAT patients at any given time (i.e., waitlists), where institutions could not induct anyone else on OAT if they had reached their operational capacity:

*“They said that there was too many people on it, and that they didn’t have enough room in the area where they did the methadone or Suboxone to add anybody else. They put me on a waiting list that never went any higher, basically. So I waited for four months and then I asked to get transferred to where I could go to get it.” (Participant 36)*

A number of the participants who were waitlisted consequently reported negative effects such as buying and using clandestine drugs, and receiving institutional charges for drug contraband during incarceration. In some cases, a few indicated they contracted infectious diseases (e.g., Hepatitis C) from sharing drug use equipment and/or overdosed during these waiting periods:

*“It did take almost a year to get on [OAT], so the wait period was very lengthy. And I did get in a lot of trouble over that year while I was trying to get on it, including that overdose.” (Participant 22)*

Additionally, some participants indicated they had used diverted OAT medications and/or other opioids/drugs during their current sentence, some of whom had done so in order to self-medicate and/or to reduce withdrawal symptoms while waiting for OAT:

*“When I came in, I went through the sickness. And I was sick probably, frigging four months, five months. And I'd still use when drugs came in … I started spending an awful lot of money … I ended up beside a cellmate and he had tons and tons of drugs. And I had a rig [syringe]. So, he started giving me drugs to use the rig. And believe it or not, that's where I ended up getting Hep C. It was in prison.” (Participant 40)*

#### Institutional and physician discrepancies

Beyond excessive waiting periods to initiate OAT, key discrepancies between institutional processes and OAT physicians were reported. Overall, nearly all participants reported that physician visits were unreliable and infrequent (commonly only once a month, and in some instances not for several months), and that waiting periods varied across institutions. This was detrimental for those who experienced problems (e.g., dosing, etc.) with their current OAT regimen and who required physician consults, but had to wait a month or more to do so. Also, participants reported inter-institutionally discrepant experiences with OAT practices and/or related interactions with health care staff. OAT processes were inconsistent, non-standardized, and appeared to be based on individual institutional policies and/or medical staff preferences and/or capabilities, rendering a diverse picture of the day-to-day operations of correctional OAT care. For example, physician’s stipulations regarding allowable dosages and time periods for increasing/reducing medications varied across institutions. Institutional OAT physicians would generally not allow the same OAT doses participants had received in the community or that they were comfortable with (i.e., physicians typically worked with set limits on OAT dose/levels). A number of participants indicated that they were using OAT not only for their OUD, but also for pain management since institutions would restrict certain pain medications, including prescription opioids. As such, many participants indicated that the OAT dose they received was insufficient to address their drug cravings, pain, and withdrawal symptoms:

*“I need a higher dose. But it’s something that I’ll have to arrange with somebody in the community, because they have a level. Like [institution name], they’ll put you up wherever you need to be, but this doctor here, he’s pretty stickler about how high.” (Participant 19)*

Furthermore, in most institutions, participants were categorically not allowed to initiate buprenorphine-naloxone unless they had a diagnosed contraindication to methadone (e.g., related to cardiovascular or other health issues). Participants further suggested that since buprenorphine-naloxone was commonly diverted, OAT physicians in some institutions would not prescribe it to OAT patients at all, or would only provide it close to an individual’s release date. Similar challenges revolved around participants’ ability to switch from methadone to buprenorphine-naloxone if they desired to do so:

*“I wanted to get off the methadone, and I told [the nurse] that I wanted to switch over to Suboxone because [it] is not as bad on your overall system … She said ‘oh, if you don’t have a heart problem, like palpitations or some kind of cardiovascular problem that the methadone causes, which I did have, um, you weren’t a candidate for going on Suboxone’. And that was that. Like you’re basically telling me I have no choice but to be a slave to the methadone.” (Participant 05)*

Another discrepancy between institutions and individual OAT physicians’ practices revolved around the specifics for switching between OAT formulations. Most physicians required participants to undergo a three-day detoxification period from methadone before they could induct buprenorphine-naloxone, which was experienced as highly undesirable due to withdrawal symptoms. However, some physicians would allow an alternative transition process through ‘micro-dosing’, where participants would slowly wean off methadone while gradually being introduced to small doses of buprenorphine-naloxone, which was more desirable. Reasons for wanting to switch from methadone to buprenorphine-naloxone included the extensive stigmatization associated with methadone, where many receiving methadone-based OAT felt ostracized and looked down upon by peers, as well as by correctional staff. It was also suggested that many individuals who would potentially benefit from OAT would not engage with it due to its stigma, and an inability to receive buprenorphine-naloxone. Generally, many participants were frustrated with the lack of personal agency and inability to freely choose the OAT formulation they felt would work best for their health and treatment:

*“When I got here I talked to the doctor about getting off methadone and going on Suboxone, and I was told that I wouldn't be able to do that until a month before I was getting released. So I always thought that the program is – you know, it should be up to you which one you want to choose.” (Participant 21)*

Several institutions were also in the process of switching from the sublingual buprenorphine-naloxone tablet to the buccal film regimen during the study as a means to mitigate diversion. Among participants who had received the new formulation, some indicated that they disliked its taste, but that it had longer-lasting effects compared to the pill format. This was primarily due to health care staff in some institutions crushing the buprenorphine-naloxone tablets before administration in order to prevent potential diversion. Many participants indicated that this procedure severely compromised the medication’s strength, resulting in subsequent withdrawal symptoms:

*“I was on Suboxone and they started crushing the pills, and when they did that, they might as well have just cut my dose in half, you know? I’m on it for pain management … it wasn’t lasting. Like whenever they just crushed it and it was like six or seven o’clock, I was starting to, you know, feel like shit.” (Participant 37)*

Another institutional discrepancy was related to the daily routine administration of OAT. Some institutions administered OAT one-on-one with participants, and would either have them come to the healthcare unit, or would go to the individual’s cell/range to deliver it, whereas other institutions utilized a mass-dosing procedure where all individuals on OAT would come to a common room daily to receive it. Most participants described the administration as onerous and lengthy, often due to staff and resource shortages and the need for nurses to monitor each participant’s dose intake for a minimum of 15 min to avoid possible medication diversion. However, most participants indicated that the OAT healthcare staff were generally helpful and supportive.

Finally, some participants discussed contempt for the OAT program, and explained that the challenges of institutional OAT care engagement, including wait times to engage in OAT care, achieving a comfortable dose, and the inability to choose their preferred OAT medication, fundamentally undermined the benefits of the program and resulted in negative health outcomes. Participants thus suggested and urged for operational changes to improve it:

*“These overdoses are happening because there’s no real alternative. The methadone program takes forever to get on, and there’s a stigma attached to it. So they should start allowing more people not only to switch from methadone to Suboxone … And then allowing more people just to join, to get on … And I think they need to find a better way to deliver it in the mornings … But a lot of those guys, they want to come off it, or they want to switch to the Suboxone … So I think that will be the biggest thing that I would change.” (Participant 25)*

#### Potential barriers to community reintegration

When considering plans and goals towards their release into the community, most participants indicated a desire to improve their health, find a job, reconnect with family and friends, and become participating members of society. Yet, participants expressed a mix of emotions such as dread, anxiety, and apprehension, combined with enthusiasm and excitement towards their impending release. Specifically, some expressed fear that they would relapse to drug (and opioid) use and end up losing support from their families and return to prison:

*“I”ve got anxiety and I'm feeling really anxious, I guess. Because in here I've got nothing but to gain [sic] towards my release, out there, I've got everything to lose. Like in here I've got my family and everything supporting me. Out there, if I go back to drug use, or crime, I come back to prison, and then it's all gone.” (Participant 15)*

Regarding their post-release plans for OAT, about four-in-five participants indicated that they planned on engaging with OAT in the community immediately after release, with nearly half of those expressing that they expected to remain on OAT for at least a year. Others indicated that while they expected to remain on OAT in the short-term, they would like to wean off OAT by the end of their parole period. However, some expressed nervousness regarding potential repercussions of ending OAT engagement prior to the end of their parole as it could conflict with formal stipulations of their correctional release plans, or be perceived as a sign of non-commitment towards addressing their substance use issues and successful community reintegration by their parole officer.

While the majority of participants indicated plans to remain engaged with OAT, they suggested several possible barriers to receiving continuous OAT care in the community, including stigma associated with OAT, the location of and lack of available transportation to OAT clinics, not having required identification/papers, difficulty in reconciling daily appointments with possible work schedules, and the possible cost of OAT care:

*“I don't know. I guess it would depend on like location maybe. I would imagine that everywhere in [city name], there's got to be pharmacies that offer Suboxone. But if I had to live by myself – apparently like Suboxone is like $20.00 a dosage every day – so if I wasn't working that could be an issue.” (Participant 15)*

Some participants described uncertainty regarding release details which they suggested made it difficult to develop concrete OAT-related care plans in advance. For participants who anticipated living in a community-based residential facility (e.g., a halfway house) or residential treatment, it was common that they would not learn of release details until shortly before, as it depended on space availability. Some indicated that not knowing these details would prevent them from securing essential community-based services and supports, and emphasized the importance of timely release planning:

*“It’s hard to picture anything without knowing where you have to put your head. You know what I mean? That’s the main thing … from there it will all fall into place. Once you know where to put your head, you can go and accumulate your stuff.” (Participant 27)*

The other major barrier to staying on OAT and accomplishing their release plans related to fears of running into past acquaintances, with half of participants expressing that their social connections and/or visiting known community OAT clinics might facilitate easy access to substances, and increase the risk for a potential relapse:

*“First thing would be going to this clinic to do my urinalysis throughout the week. I’m worried because I’ve lived in [city name] my entire life and I know a lot of people and part of my problem is accessibility to drugs. So if I’m going there every day it’s only a matter of time before I start running into old acquaintances, people that I know, and they might have access to drugs for me.” (Participant 22)*

#### Potential facilitators to community reintegration

Participants also indicated a few key potential facilitating factors that they suggested would help them reach their goals and effectively continue OAT after release. Securing a job and housing were among the top suggestions, as was the ability to receive OAT ‘carries’ (i.e., take-home doses of OAT medication) as quickly as possible so that they would be more flexible and able to balance their work or other responsibilities against the (e.g., daily) rigorous clinic attendance requirements to obtain their OAT medications. Other potential facilitators included access to substance use-specific treatments and support programs.

When asked if there was anything in particular that would support their post-release transition and allow them to reach their goals, the most common response - indicated by about two-in-three participants - was familial support. Examples included relying on family members to drive them to their OAT and other appointments, as well as for housing, social, and financial support:

*“My mum said if I have to get to the pharmacy or whatever, daily, like she would make sure I get there. Because I’m not allowed to drive. And I probably will be in, like counselling. And hopefully I’m on this Suboxone program and I can attain carries shortly after, so that I can continue the program without the daily interruption of appointments and the pharmacy.” (Participant 22)*

Lastly, some participants expressed that one of the most important facilitators for community reintegration was personal motivation. Many participants spoke about how they had finally reached a point where they were determined and ready to commit to staying abstinent.

## Discussion

This longitudinal, mixed-methods study examined experiences with opioid use, community- and institutional-based OAT, and related perspectives on community release among a sample of federally incarcerated individuals in correctional institutions in Ontario, Canada. Consistent with other studies involving correctional populations, the results indicated that most participants had self-reported mental health and substance use problems, as well as a longstanding and complex history of drug - and specifically opioid - use and related issues [1, 71]. Opioid use trajectories commonly included quick transitions to high-intensity opioid use, including frequent (e.g., daily) use of strong (e.g., fentanyl) opioids used intravenously, which negatively affected participants’ health, and in some cases was a contributing cause of their arrest and current incarceration. Additionally, participant experiences with OAT varied, both in the community as well as during incarceration, which shaped their perspectives on its efficacy, utility, and expectancies related to OAT engagement upon release into the community [2, 48]. Furthermore, notable characteristics of the study sample include a high proportion of young men (a third of whom were Indigenous) with high-risk (e.g., injection, sharing equipment, overdose, etc.) drug use patterns and histories, yet relatively short sentences. These characteristics are unsurprising given the longstanding impacts of drug prohibition in Canada and the specific criminalization of illicit drug use which has disproportionately impacted marginalized populations and contributed to the overrepresentation of Indigenous peoples in the Canadian correctional system [12, 72–74]. These factors point to an increased risk of substance use and health-related impacts both during incarceration and post-release. However, participants expressed that OAT was beneficial in terms of reducing drug cravings and use, as well as towards meeting their goals post-release. This implies the opportunity and need for adequate OAT treatment initiation and related care during incarceration, as well as continuous treatment provision during and throughout the community transition period.

As the study results substantiate, drug use prior to and during incarceration is common, despite institutional policies prohibiting contraband from entering institutions [11, 15–18, 75]. Participants detailed persistent substance use and related issues during incarceration, including as a means of pain management and to combat withdrawal symptoms, especially when they experienced OAT disruptions [76, 77]. Regarding OAT initiation and provision during incarceration, a number of key access barriers were reported. These are largely in line with existent literature highlighting programmatic, attitudinal, and systemic barriers to correctional institution-based OAT and healthcare provision. These include a lack of professional skills, capacity, and resources (including qualified staff), restrictive policies, logistical obstacles such as institutional movement schedules, security concerns including OAT diversion, and a general lack of support and resources for treatment-oriented care in an environment generally shaped by risk reduction [29, 31, 78–80].

Importantly, the most common barrier reported related to extensive waiting periods regarding resuming or initiating institution-based OAT care during which time participants were at risk of experiencing withdrawal symptoms and overdose events, which they commonly had to endure with limited clinical management or oversight. These issues were sometimes aggravated by individuals entering federal correctional institutions through provincial correctional systems first (e.g., individuals commonly present to provincial correctional institutions upon arrest and are held in remand while they await sentencing prior to transferring to federal institutions), where OAT prescribing practices and polices are diverse and non-standardized [33]. These issues have been identified by a recent human rights complaint [81], but they also categorically conflict existing clinical guidelines for community-based OAT (which does not recommend withdrawal management as a safe or appropriate treatment option), and contravene standing recommendations for seamless access to OAT for Ontario-based correctional populations [21, 33, 82]. As such, it can be seen as imperative that both provincial and federal correctional institutions consistently screen for OUD upon admission and actively work towards eliminating waitlists to ensure that all individuals who require OAT have immediate access, and do not need to undergo unnecessary withdrawal and related discomforts and risks for serious adverse health outcomes [83, 84].

Our results also demonstrated that even while national federal correctional (CSC) guidelines on institutional OAT provision exist [20], implementation differs across institutions (e.g., in regards to dosing, administration, processes, formulations, physician practices, etc.). Many physicians limited access to buprenorphine-naloxone in particular despite participants’ preference for this OAT formulation due to its perceived benefits. Other qualitative studies have suggested comparable preferences for buprenorphine-naloxone over methadone among both community and correctional-based populations, with methadone often linked to more negative health experiences and perceptions [85–89]. Furthermore, participants described methadone as being particularly stigmatized, which may have impacted individual’s decisions around OAT engagement. For instance, participants described experiencing negative judgments for being engaged in methadone, yet experienced major access barriers to buprenorphine-naloxone, which may have kept individuals with OUD from engaging in OAT altogether, and/or influenced those who could not access buprenorphine-naloxone to withdraw from treatment, rendering them vulnerable to related health harms [37, 90, 91]. Many of these restrictive practices seem to arise from organizational policies and structures that prioritize punishment and risk-reduction (e.g., diversion) over health concerns or care. As such, participants described a detrimental lack of agency and autonomy in regards to their OAT care. Such patient-centered care has been strongly advocated for and associated with improved outcomes, particularly for individuals with substance use problems, including OUD [92–95].

These factors highlight the need for a number of critical policy and operational improvements in the correctional system, including the need to standardize OAT provision and processes across institutions so that individuals can expect the same level of care that they would receive in the community [96]. Institutions should also expand access to a variety of evidence-based OAT formulations such as extended-release injectable buprenorphine (i.e., Sublocade) or antagonists (such as Naltrexone) which have been associated with positive outcomes among correctional populations and would improve patient-centered care [97–102]. Telehealth/telemedicine is another alternative approach to conventional care and a potential solution to improve access and quality of health care [103, 104]. These recommendations are warranted in the context of the ‘epidemic’ of opioid-related overdoses which has unfolded both across Canadian communities and correctional systems [19, 105–107]. While many of these OAT options are new and evidence on their effectiveness and feasibility among correctional populations is limited, literature points to positive outcomes [100, 108–112].

Notably, a recent internal evaluation of CSC’s healthcare services recommended improvements to the delivery, content, and monitoring of OAT programs, as well as the provision of adjunct addictions counseling, health-oriented education, and harm reduction programming [53]. Since this evaluation – and the present study’s data collection – CSC has further accelerated the roll-out of buprenorphine-naloxone as a first-line treatment option. Buprenorphine-naloxone-based OAT numbers are increasingly outpacing methadone-based OAT at many institutions [113]. Specifically, by January 2021, over half (62%) of the total 2481 individuals engaged in OAT in CSC institutions were receiving buprenorphine-naloxone, and 190 were receiving extended-release injectable buprenorphine [46]. Furthermore, a number of participants described using OAT for pain management, particularly as institutions would restrict certain medications (e.g., prescribed opioids). Since OAT is not necessarily meant for pain management, this has important implications for treatment programming, and underscores the need for individuals to have access to appropriate care (including pharmacotherapy) [26, 96]. CSC is in the process of developing chronic non-cancer pain management guidelines which should help this issue [114]. These practicalities may address some of the OAT-related issues identified by our study participants.

With regard to participants’ plans and perceived barriers and facilitators of community OAT transitions upon impending community release, the study’s findings corroborate many issues documented by other studies among recently-released individuals with OUD. These include fear of exposure to drugs through peer networks resulting in the likelihood of relapse, financial, employment and housing instability, logistical (e.g., transportation) issues, and other barriers to effective community-based treatment continuation [88, 89, 115–119]. Furthermore, participants indicated that a lack of clarity around their impending release details contributed to anxiety and  an inability to adequately plan for OAT care and general reintegration. These observations underscore the need for timely, consistent, and collaborative community release planning and case management that takes personal readiness/motivation and key determinants of release outcomes into consideration towards setting individuals up for improved community reintegration and success [120]. Participants also expressed that an important facilitator to successful community reintegration was familial support; meanwhile, they indicated a main barrier would be returning to places where their families reside, possibly increasing the risks of exposure to deviant social networks and re-accessing or relapsing to substance use. While balancing these two factors may be difficult, many participants alluded that the critical factor would be personal motivation and/or self-efficacy, and that through CSC-based correctional programming (e.g., structured interventions that target risk factors directly linked to criminal behaviour in order to reduce reoffending) [121], they had learned the skills to combat their triggers and cravings, abstain from drug use, and continue with OAT care.

Overall, this study highlighted a variety of issues related to the administration of OAT within Ontario-based federal correctional institutions. However, results also indicate that OAT is beneficial and supports the reduction of opioid use and cravings among individuals with OUD, which will ideally translate into improved health, and more effective and successful post-release community reintegration. Importantly, correctional institutions offer a unique opportunity to provide OAT treatment for individuals with complex substance use histories, including OUD, who may otherwise not engage in treatment [80, 122, 123]. In addition, since most federally incarcerated individuals experience a period of community-based supervision which both allows and requires them to re-integrate into society, it is an opportune time to ensure continuous linkage of OAT and other necessary health and social supports during this high-risk transitional period [2].

Possible study limitations ought to be noted. While the study results reflect the participants’ self-reported experiences, they should be interpreted with appropriate caution as they may include inherent biases in self-report data (e.g., memory/recall/response bias, interpretation of questions, and social desirability). Additionally, participants may have had unconscious negativity biases that resulted in a tendency to report on and remember negative impacts and experiences over positive ones. These biases may be especially applicable to correctional study settings and/or populations, and related strict behavioral norms and/or potential repercussions related to rule-breaking (e.g., drug use, contraband, etc.). As such, participants may have downplayed their involvement in drug use or deviant institutional activities; at the same time, the study likely obtained an authentic empirical picture given its execution by an outside team independent of possible institutional consequences (e.g., coercion). The results are not generalizable, for CSC or other (including provincial) correctional populations as the study sample was small and cannot be considered representative. Study criteria to include those who had been in OAT care for at least 3 months was chosen to ensure individuals were stabilized; however, the sample and results might therefore have differed from those who were on the program for less than 3 months. Those who were screened and did not meet eligibility may have also differed in significant ways from the actual study sample. Moreover, the sub-sample of women was especially small, rendering an inability to conduct any sex/gender comparisons. Only around 6% of federally incarcerated individuals in Canada are women, and there is only one women’s prison in Ontario. As such, the limited number of women participants included was to be expected. However, similar sentiments towards OAT were provided by both sexes. Sex/gender differences should be better and systematically examined by future research.

## Conclusions

The prevalence of OUD, and the need for effective OAT-based care in the Canadian federal correctional system is high. While general OAT access has expanded over-time, distinct structural and organizational factors appear to hinder OAT uptake and have resulted in adverse health and social outcomes. The study’s results point to a number of concrete recommendations. These include the elimination of waitlists, the standardization of OAT processes across institutions, a focus on patient-centered care that puts individuals at the forefront of their treatment decisions, the expansion of OAT formulations and telehealth care, and the need for more robust discharge planning to ensure individuals are adequately prepared for community release. Federal correctional institutions and policy makers should consider these suggestions in order to tangibly improve on the design, delivery, and practices of OAT care in Canadian prisons.

## Figures and Tables

**Fig. 2:**
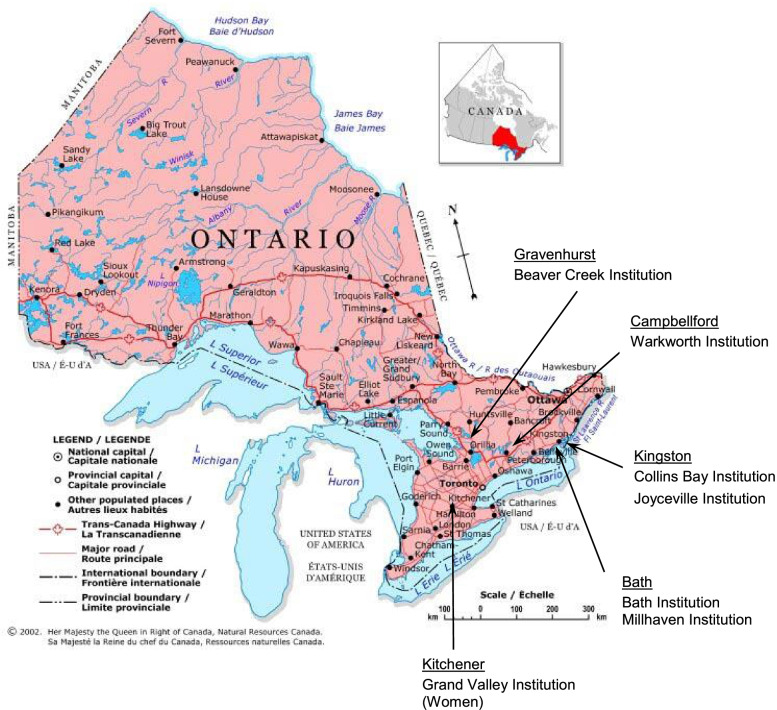
MAP

## Data Availability

The datasets generated and/or analyzed during the current study are not publicly available due to participants not providing consent to share their data beyond the research team, but are available from the corresponding author on reasonable request**.**

## Appendix 1

Table 4

**Table 4:** Map and security classification levels of federal Correctional Service Canada institutions in Ontario

Institution	Security Classification Levels
Bath Institution	Medium Security Level
Beaver Creek Institution	Medium and Minimum Security Levels
Collins Bay Institution	Maximum, Medium and Minimum Security Levels
Grand Valley Institution for Women	Multi Security Levels
Joyceville Institution	Medium and Minimum Security Levels
Millhaven Institution	Maximum Security Level
Warkworth Institution	Medium Security Level

## Appendix 2

### Qualitative Interview Guide

#### Qualitative questions

(NOTE: these questions and responses will be audiotaped)

24. Please **DESCRIBE** your experiences with **OPIOID USE** and **DEPENDENCE PRIOR** to your **CURRENT** incarceration period

**Prompts:**

○ How did your opioid use begin?

○ Has your opioid drug use changed over time?

○ Has opioid drug use impacted the following areas of your life: Health? Relationships? Work/Finances? Housing?

25. Please **DESCRIBE** your experiences with **OST PRIOR** to your **CURRENT** incarceration period

**Prompts:**

○ If you were engaged in OST prior to incarceration, how was the experience? What factors made the experience good or bad?

○ If you were not engaged in OST prior to incarceration, why not?

26. Please **DESCRIBE** your experiences with **OPIOID** and **OTHER DRUG USE DURING** your **CURRENT** incarceration period

**Prompts:**

○ Which opioids are available? What other drugs are available? Tell me about this.

○ Aside from your OST, which opioid-related supports/services are available? Which other drug use-related supports/services are available?

○ Have you used these supports/services? If so, tell me about your experiences with these.

27. Please **DESCRIBE** your experiences with **OST DURING** your **CURRENT** incarceration period

**Prompts:**

○ How did you first access OST? Tell me about your experience.

○ Are there any factors have been helpful to you? Is there anything you would change?

28. Please **DESCRIBE** your **PERSONAL GOALS/PLANS** (e.g., related to health, relationships, work/finances, housing) for your upcoming **RELEASE** into the community.

**Prompts:**

○ What are your general feelings towards your upcoming release into the community?

○ Is there anything you are looking forward to?

○ Is there anything you are worried about?

29. Please **DESCRIBE** your **DRUG USE** and **OST**-related **GOALS**/**PLANS** for your upcoming **RELEASE** into the community

**Prompts:**

○ What do you expect will happen with your opioid and/or other drug use following release?

○ What do you expect will happen with your OST following release?

30. Please **DESCRIBE** what **FACTORS**/**SUPPORTS** you see as particularly **IMPORTANT** in achieving your **DRUG USE** and **OST**-related goals and plans upon **RELEASE** into the community

**Prompts:**

○ Is there something you feel is *particularly* important for helping you reach your drug use-related goals?

○ Is there something you feel is *particularly* important for helping you reach your OST-related goals?

31. Please **DESCRIBE** any potential **CHALLENGES**/**BARRIERS** you foresee towards achieving your **DRUG USE** and **OST**-related goals and plans upon **RELEASE** into the community

**Prompts:**

○ Is there something you feel may make it *particularly* difficult to reach your drug use-related goals?

○ Is there something you feel may make it *particularly* difficult to reach your OST-related goals.

32. Please **DESCRIBE** where you see yourself a **YEAR** after your **RELEASE** into the community

**Prompts:**

○ Specifically regarding your opioid and/or other drug use?

○ Specifically regarding your OST?
